# Supplementary material for: MetaRibo-Seq measures translation in microbiomes
Source: Nat Commun. 2020 Jun 29;11:3268. doi: 10.1038/s41467-020-17081-z (PMC7324362; doi:10.1038/s41467-020-17081-z)
Supplement: Supplementary file 10 — Supplementary Data 7 [file 41467_2020_17081_MOESM10_ESM.zip › File2/Confidence_High_Taxonomy/202881_out.krona.html]

Javascript must be enabled to view this page.

members
magnitude
magnitudeUnassigned
count
unassigned
taxon
rank

202881\_out

56

56
2
superkingdom

56
1239
phylum

1262992
56

SRS013638\_contig\_number\_16268SRS013940\_contig\_number\_13528SRS014683\_contig\_number\_contig-100\_13178.61407SRS014923\_contig\_number\_37574SRS015217\_contig\_number\_20929SRS016495\_contig\_number\_17171SRS017521\_contig\_number\_29539SRS018427\_contig\_number\_16523SRS018936\_contig\_number\_8559SRS019030\_contig\_number\_contig-100\_30929.30929SRS019068\_contig\_number\_contig-100\_13765.315283SRS019601\_contig\_number\_contig-100\_23759.103521SRS020622\_contig\_number\_1033SRS022137\_contig\_number\_1396SRS023715\_contig\_number\_27740SRS024492\_contig\_number\_contig-100\_6780.81111SRS024663\_contig\_number\_contig-100\_4187.67535SRS042628\_contig\_number\_24597SRS045826\_contig\_number\_7825SRS046502\_contig\_number\_6116SRS050925\_contig\_number\_1278SRS063518\_contig\_number\_30897SRS074964\_contig\_number\_contig-100\_6785.6785SRS075821\_contig\_number\_contig-100\_30079.80297SRS075984\_contig\_number\_contig-100\_7701.88475SRS076756\_contig\_number\_10475SRS076976\_contig\_number\_2053SRS077194\_contig\_number\_contig-100\_9707.119861SRS077641\_contig\_number\_6697SRS097920\_contig\_number\_24371SRS098571\_contig\_number\_13443SRS1041037\_contig\_number\_5391SRS1041118\_contig\_number\_contig-100\_11609.11610SRS1041138\_contig\_number\_7631SRS104327\_contig\_number\_361SRS104912\_contig\_number\_contig-100\_871.124898SRS1055043\_contig\_number\_contig-100\_559.155044SRS1055099\_contig\_number\_contig-100\_13828.13829SRS140492\_contig\_number\_11442SRS143148\_contig\_number\_contig-100\_17301.101729SRS143372\_contig\_number\_contig-100\_1041.1042SRS143417\_contig\_number\_3679SRS143523\_contig\_number\_16294SRS143722\_contig\_number\_contig-100\_9892.43329SRS143991\_contig\_number\_21618SRS144506\_contig\_number\_51068SRS146764\_contig\_number\_30206SRS147377\_contig\_number\_contig-100\_11530.11531SRS148253\_contig\_number\_13588SRS148721\_contig\_number\_contig-100\_4214.261858SRS148817\_contig\_number\_contig-100\_17366.57064SRS149075\_contig\_number\_9742SRS893292\_contig\_number\_contig-100\_21319.21320SRS893358\_contig\_number\_contig-100\_1835.39562SRS893378\_contig\_number\_5903SRS971275\_contig\_number\_contig-100\_11974.123289
species
